# Effect of Academic Detailing on Promoting Appropriate Prescribing of Antipsychotic Medication in Nursing Homes: A Cluster Randomized Clinical Trial

**DOI:** 10.1001/jamanetworkopen.2020.5724

**Published:** 2020-05-26

**Authors:** Mina Tadrous, Kinwah Fung, Laura Desveaux, Tara Gomes, Monica Taljaard, Jeremy M. Grimshaw, Chaim M. Bell, Noah M. Ivers

**Affiliations:** 1 ICES, Toronto, Ontario, Canada; 2 Women’s College Research Institute, Women’s College Hospital Institute for Health Systems Solutions and Virtual Care, Women’s College Hospital, Toronto, Ontario, Canada; 3 Institute of Health Policy, Management, and Evaluation, University of Toronto, Toronto, Ontario, Canada; 4 Leslie Dan Faculty of Pharmacy, University of Toronto, Toronto, Ontario, Canada; 5 Clinical Epidemiology Program, Ottawa Hospital Research Institute, Ottawa Hospital, Ottawa, Ontario, Canada; 6 University of Ottawa School of Epidemiology, Public Health and Preventive Medicine, Ottawa, Ontario, Canada; 7 Clinical Epidemiology Programme, Ottawa Hospital Research Institute, Ottawa, Ontario, Canada; 8 Department of Medicine, Mount Sinai Hospital, Toronto, Ontario, Canada; 9 Department of Family and Community Medicine, Unviersity of Toronto, Toronto, Ontario, Canada

## Abstract

**Importance:**

Strategies to reduce the inappropriate prescription of antipsychotics have been the focus of recent attention but have shown considerable variation in their effectiveness.

**Objective:**

To evaluate the effectiveness of academic detailing in nursing homes targeting appropriate prescribing of antipsychotics.

**Design, Setting, and Participants:**

We conducted a pragmatic, cluster randomized clinical trial comparing the effect of academic detailing vs usual care on prescribing antipsychotics in 40 nursing homes with 5363 residents in Ontario, Canada. Data were collected from October 2015 to March 2016 and analyzed from April to August 2018. Primary analyses were conducted using intention to treat.

**Intervention:**

Academic detailing delivered by health professionals (eg, nurses or pharmacists) who arranged meetings (with administrators, physicians, pharmacists, nurses, and support workers), presentations, group visits (with 2-6 clinicians), and 1-on-1 visits (traditional academic detailing visits). Academic detailers had direct and ongoing contact with the nursing homes from the time of launch.

**Main Outcomes and Measures:**

The primary outcome, defined at the level of the resident, was whether continuous antipsychotics were dispensed in the past week. Secondary outcomes included prescribing of other psychotropic medications and clinical outcomes and scores. Prescribing outcomes were assessed at baseline and at 3, 6, and 12 months, and clinical outcomes and scores were assessed at baseline and 3 and 6 months.

**Results:**

A total of 40 nursing homes with 5363 residents were randomized, with 18 intervention homes (45.0%; 2303 [42.9%] residents) and 22 control homes (55.0%; 3060 [57.1%] residents). Participants in the intervention and control groups had similar median (interquartile range) age (86 [79-91] years vs 85 [78-90] years) and sex (674 [29.3%] men vs 970 [31.7%] men). At 12 months, there was no statistically significant difference in the frequency of daily antipsychotic use (intervention: 569 patients [25.2%]; control: 769 [25.6%]; odds ratio, 1.06; 95% CI, 0.93-1.20; *P* = .49). There were no significant differences in the rates of health care utilization, but the intervention group did experience a statistically significant reduction in pain compared with the control group (mean [SD] pain score, 0.30 [0.59] vs 0.38 [0.66]; *P* < .001) and depression (mean [SD] Depression Rating Scale score, 2.18 [2.37] vs 2.81 [2.65]; *P* < .001) at 6 months.

**Conclusions and Relevance:**

The intervention did not further reduce antipsychotic prescribing in nursing homes beyond system-level secular trends occurring alongside usual care. Our findings highlight the need for a more targeted approach to quality improvement strategies, including academic detailing, that account for the timing and topic of interventions.

**Trial Registration:**

ClinicalTrials.gov Identifier: NCT02604056

## Key Points

**Question:**

What is the real-world effectiveness of an academic detailing intervention in nursing homes to improve the appropriate prescribing of antipsychotics?

**Findings:**

In this cluster randomized clinical trial of 40 nursing homes with 5363 residents in Ontario, Canada, academic detailing did not further reduce antipsychotic prescribing beyond a downward secular trend.

**Meaning:**

The results of this study suggest that academic detailing in nursing homes may not be an effective intervention to reduce antipsychotic prescribing in an environment where standard quality improvement supports are working.

## Teaser

This cluster randomized clinical trial evaluates the effectiveness of an academic detailing intervention in nursing homes to improve appropriate prescribing of antipsychotic medications.

## Introduction

Potentially inappropriate use of antipsychotics in nursing homes has been the focus of significant clinical, research, and policy attention.^1,2,3,4^ The increased use of antipsychotics during the early 2010s raised concern because of its questionable effectiveness and a well-established risk profile.^5,6^ Regulators have issued numerous warnings related to increased harm associated with this drug class, including higher mortality among elderly patients prescribed antipsychotics.^7,8,9,10,11^

In 2013, approximately 35% of nursing home residents in Ontario were prescribed antipsychotics, compared with only 4.6% of seniors living in the community.^12^ Similar results have been observed in many jurisdictions internationally.^13,14,15^ Strategies to reduce inappropriate prescription of antipsychotics have been shown to have considerable variation in their effectiveness.^16,17^ Many trials of these interventions have short-term follow-up (ie, <6 months) and are often small in scale. The objective of this study was to evaluate the real-world effectiveness of an academic detailing intervention in nursing homes across Ontario targeting appropriate prescribing of antipsychotics and the management of behavioral and psychological symptoms of dementia.

## Methods

### Trial Design

Details of the trial design and rationale have been reported previously,^18^ and the study protocol is available in Supplement 1. Briefly, we conducted a pragmatic cluster randomized clinical trial in nursing homes that shared physicians as the unit of randomization.^19,20^ An independent statistician randomly allocated the homes using computer-generated random numbers. Randomization occurred once at the start of the study. Nursing homes were allocated to the full, active intervention (ie, academic detailing) or standard quality improvement supports (ie, usual care). Due to the nature of the intervention, masking of group allocation was not possible. An embedded process evaluation was conducted to explore why the intervention did or did not work and is described elsewhere.^21,22^ The evaluation was led by an team of academics, who developed the methods in collaboration with the partners responsible for sponsoring and implementing the interventions, namely the Ontario Ministry of Health and Long-term Care, the Ontario Medical Association, the Centre for Effective Practice, and Health Quality Ontario. For logistic reasons, the academic detailing intervention was administered in 2 waves (October 2015 and February 2016), each for 6 months. The results presented in this article describe homes included in the first wave because the second wave was not randomized to have controls. The second wave was completed based on remaining funding for the program that allowed an extension of the service to additional homes.

The study received ethics approval from the Women’s College Hospital and the University of Toronto Research Ethics Boards. The entire nursing home was considered a research participant, so written informed consent was obtained from the medical director and administrative leads from each home. This study followed the Consolidated Standards of Reporting Trials (CONSORT) reporting guideline for cluster randomized trials.^23^

### Participants

Homes were eligible if they were a reasonable travel distance (ie, <100 km) from the central intervention team in the greater Toronto area. Identification of potential homes was completed in collaboration with stakeholders, including the Ontario Long-term Care Association and AdvantageAge Ontario. To be eligible for participation, the medical and administrative leads of the homes were required to express support. Exclusion criteria were homes without any prescribers caring for at least 10 residents at baseline or homes with fewer than 30 residents. A recruitment email was distributed by the Ontario Ministry of Health and Long-term Care to administrative leads and medical directors of nursing homes. Approval for trial participation was obtained from the medical director and administrative leads from each home. Individual clinicians and staff members in the home were free to independently decide whether and how to participate in the intervention.

### Intervention

#### Academic Detailing

Academic detailing is a method of educational outreach that leverages 1-on-1 interactions to communicate evidence-based information to clinicians.^24^ The intervention was delivered by health professionals (eg, nurses or pharmacists) who arranged meetings (with administrators, physicians, pharmacists, nurses, and support workers), presentations, group visits (with 2-6 clinicians), and 1-on-1 visits (traditional academic detailing visits). Academic detailers had direct and ongoing contact with the nursing home staff, including administrators, clinicians, and staff from the time of launch to program completion. Detailers provided tailored information to help to support nursing home clinical staff to address challenges and opportunities for improving prescribing of antipsychotics, providing additional materials and resources as needed. They also responded to questions via email or telephone. Given the diverse target audiences that would likely be engaged in the homes, the academic detailer worked to understand the context, barriers, capacity, and needs of each home to ensure the service being provided was relevant.

#### Usual Care

Usual quality improvement supports included confidential physician-level online report cards that described their prescribing practices for antipsychotics compared with regional and provincial rates, along with clinical and demographic features of the physician’s roster. Physicians voluntarily signed up and confirmed their identity to receive updated quarterly reports, which have been available province-wide since October 2015. The reports are produced by Health Quality Ontario using data from ICES, a nonprofit research institute that holds Ontario’s health-related data. In addition to the reports, virtual communities of practice were launched to allow sharing of best practices across the province.

### Data Sources

Outcomes were assessed using the following population-level administrative claims databases, linked through unique identifiers available at ICES, as follows: (1) the Ontario Drug Benefits database, covering all publicly funded prescription medications dispensed to residents in nursing homes; (2) the Canadian Institute for Health Information databases, covering all inpatient hospitalizations and emergency department visits; (3) the Ontario Health Insurance Plan database, covering physician billings for procedures and consultations; (4) the Registered Persons database, covering demographic information, including date of death; and (5) the Continuing Care Reporting System (CCRS) database for clinical and demographic information on nursing home residents collected using the Resident Assessment Instrument (RAI) (eTable 1 in Supplement 2). Legislation requires that each nursing home resident in the province receive a full RAI assessment within 2 weeks of their admission date, every 12 months from previous full assessment, or any time their clinical condition changes considerably. Furthermore, each resident also receives a shorter, quarterly assessment. The CCRS database contains information on diagnoses (eg, dementia), dispensing of antipsychotics, and incidence of falls, among other data. Additionally, a number of validated outcome scores are available from the CCRS, including those related to activities of daily living, aggressive behaviors, pain, and mood.^25,26,27,28^

### Outcomes

The primary outcome was antipsychotic medication dispensed, defined at the level of the resident. This was operationalized as the number of days the resident was provided an antipsychotic in the previous week based on the most up-to-date RAI assessment, as reported in the CCRS, and it was dichotomized prior to analysis as continuous prescription for 7 days or not. Dichotomization was completed because it was found that 99% of reporting was either 0 or 7 days. Nursing home staff complete RAI assessments within 14 days of admission, and assessments are updated annually or with a change in status; a quarterly RAI assessment is required every 92 days. Secondary resident-level prescribing outcomes included mean daily antipsychotic dose (during the previous month) and any antipsychotic, benzodiazepine, antidepressant, mood stabilizer, and acetaminophen prescribing (defined as any prescription during the previous month), determined from dispensing information. Daily antipsychotic doses were calculated by converting all active antipsychotic prescriptions into chlorpromazine daily dose equivalents. Primary and secondary prescribing outcomes were assessed at baseline and 3, 6, and 12 months after randomization. Baseline was defined as the week or month immediately before randomization, while postintervention measurements were defined as the third, sixth, and twelfth months after randomization. At each point, outcomes were assessed only for residents alive at the time of the assessment and present in the home for the full duration of the prior month. This analysis included all residents of the home, even if they were not residents at baseline. Secondary resident-level clinical outcomes included the number of falls in the past month, measured as the number of emergency department visits and hospitalizations during the past month, and clinical scores, specifically the Activities of Daily Living (range, 0-24), Aggressive Behavior Scale (range, 0-12), pain score (range, 0-3), and Depression Rating Scale (range, 0-14).^29,30,31,32,33^ These were assessed at baseline and at 3 and 6 months postrandomization. The data collected immediately before the implementation of the intervention and at each postrandomization point was used, regardless of a participant’s mortality status at the subsequent point. We also reported the number of visits and presentations made overall and per home.

### Sample Size

The a priori sample size target was determined based on logistical constraints. Specifically, the funding specified that a maximum of 40 homes would receive academic detailing in 2 waves. Only the first wave was randomized in our study. For our primary outcome (ie, days with an antipsychotic prescription in the past 7 days), we required 45 independent clusters to achieve an 80% power to detect a minimally important difference of 0.6, assuming 120 residents per home and an intraclass correlation coefficient of 0.01. For secondary prescribing outcomes using a simulation study with 1000 simulation runs, a power of 83.4% to detect an odds ratio (OR) of 0.75 at 6 months was calculated.

### Statistical Analysis

Descriptive statistics were calculated for all variables. Continuous variables with a normal distribution were described using means and SDs, categorical variables were summarized using frequencies and proportions. Primary analyses were conducted with an intention-to-treat analysis. We used a resident-level, repeated measures analysis with generalized linear mixed-effects regressions. Dichotomous outcomes were analyzed using a binomial distribution and logit links; continuous outcomes, using normal distribution and identity link. The model included fixed effects of time (categorical), intervention, and intervention-by-time as the main variables of interest, plus random effects for the home to account for clustering. To account for within-period and between-period correlation within homes, random intercepts and period effects were included, defined at the level of the home. An additional random effect was used to account for repeated measures on the same resident over time. Prespecified secondary analyses were conducted to explore prescribing trends in the year before and after the intervention. This secondary analysis was included with our initial plan to allow more time for the intervention to have an effect, based on findings in our process evaluation.^21^ All analyses were completed using SAS version 9.4 (SAS Institute) at ICES in Toronto, Ontario. Statistical significance was set at *P* < .05, and all tests were 2-tailed.

## Results

**Figure 1.**
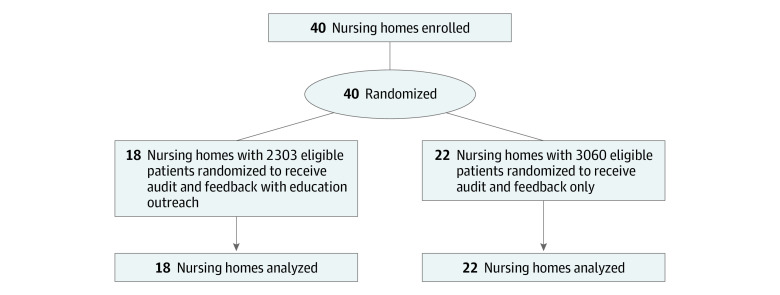
Study Flow Diagram for Cluster Randomized Trial

A total of 40 nursing homes were randomized, 18 (45.0%) to intervention and 22 (55.0%) to usual care (Figure 1). During the intervention, there were 336 clinicians engaged with a mean (range) of 8.2 (0-36) visits per home (eTable 2 in Supplement 2). There were 112 presentations and meetings held, with a mean (range) of 6.2 (1-14) per home. A total of 5363 patients (2303 [42.9%] in the intervention group; 3060 [57.1%] in the control group) resided in the homes at baseline. The intervention and control groups of the study had similar median (interquartile range) age (86 [79-91] years vs 85 [78-90] years) and sex (674 [29.3%] men vs 970 [31.7%] men). Homes were well balanced at baseline across most home-level and resident-level characteristics, except for a slightly higher percentage of residents with a history of psychosis in the usual care group than the intervention group (613 [20.0%] vs 365 [15.8%]) (Table 1). Both groups also had a similar proportion of patients living with a diagnosis of dementia (intervention: 2046 [88.8%]; control: 2708 [88.5%]).

**Table 1.** Baseline Characteristics of Nursing Homes and Residents

Characteristic	No. (%)	
	Intervention (n = 18)	Control (n = 22)
Nursing home characteristics		
Residents per home, median (IQR), No.	138 (86-168)	126 (63-168)
Beds, median (IQR), No.	153 (97-192)	145 (96-246)
Private ownership	13 (72.2)	16 (72.7)
Nonprofit ownership	5 (27.8)	6 (27.3)
Urban location	13 (72.2)	17 (77.3)
Patient characteristics at baseline		
Residents, No.	2303	3060
Age, y		
Median (IQR)	86 (79-91)	85 (78-90)
<66	162 (7.0)	236 (7.7)
66-74	213 (9.2)	322 (10.5)
75-84	603 (26.2)	875 (28.6)
≥85	1325 (57.5)	1627 (53.2)
Men	674 (29.3)	970 (31.7)
Dementia diagnosis	2046 (88.8)	2708 (88.5)
Alzheimer disease	212 (9.2)	211 (6.9)
Vascular dementia	34 (1.5)	50 (1.6)
Dementia in other disease or unspecified	2044 (88.8)	2707 (88.5)
Psychosis	365 (15.8)	613 (20.0)
Concurrent drugs, median (IQR), No.	7 (5-10)	7 (5-10)
Residents hospitalized	1084 (47.1)	1451 (47.4)
Hospitalizations in previous 3 y		
0	227 (9.9)	364 (11.9)
1	418 (18.2)	524 (17.1)
≥2	574 (24.9)	721 (23.6)
Visits in 3 mo before baseline		
Psychiatrist	76 (3.3)	144 (4.7)
Geriatrician	39 (1.7)	31 (1.0)
Neurologist	30 (1.3)	57 (1.9)
Hospitalizations in 6 mo before baseline	268 (11.6)	350 (11.4)
ED visits in 6 mo before baseline	342 (14.9)	463 (15.1)

Abbreviation: ED, emergency department; IQR, interquartile range.

**Figure 2.**
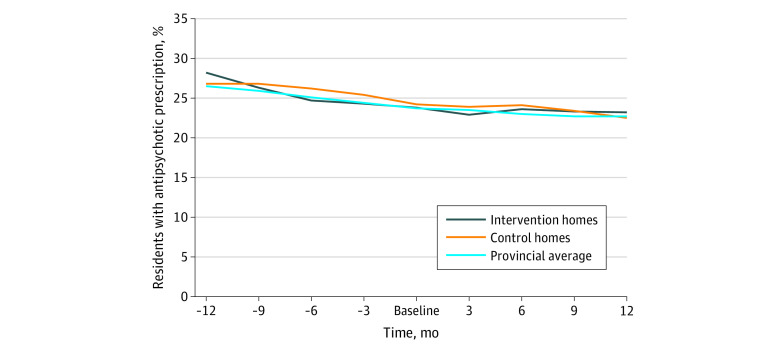
Proportion of Residents Receiving Any Antipsychotic Prescription 1 Year Before and After Intervention

The proportion of residents receiving an antipsychotic prescription decreased over time in both the intervention (522 [28.2%] to 539 [23.2%]) and control (678 [26.8%] to 694 [22.5%]) groups during a 24-month period (ie, in the 12 months before and the 12 months after randomization). These appear to parallel a similar reduction observed across the province (8727 of 32 992 [26.5%] to 9109 of 40 202 [22.7%]) (Figure 2).

At baseline, the number of residents with daily antipsychotic use in the past 7 days was 559 (25.0%) in the intervention group and 805 (27.0%) in the usual care group (*P* = .84) (Table 2). At 12 months, the number of residents with daily antipsychotic use in the past 7 days was 569 (25.2%) in the intervention group and 769 (25.6%) in the usual care group. The between-group difference and between-group difference in change from baseline to 12 months were not statistically significant (OR, 1.06; 95% CI, 0.93-1.20; *P* = .49) (Table 2). Similarly, the change in proportion of residents with a continuous daily prescription in the past 28 days was not significantly different between groups (OR, 0.88; 95% CI, 0.65-1.22; *P* = .66).

**Table 2.** Primary and Secondary Prescribing Outcomes

Outcome	Participants, No. (%)		Intervention effect from baseline (95% CI)^a^	*P* value
	Intervention (n = 2303)	Control (n = 3060)		
**Primary outcome: daily antipsychotic use in the past 7 d**				
Baseline	559 (25.0)	805 (27.0)	NA	.84
3 mo	565 (25.0)	787 (26.4)	1.02 (0.92 to 1.13)	.90
6 mo	574 (25.6)	795 (27.0)	1.02 (0.91 to 1.14)	.91
9 mo	559 (24.7)	780 (25.8)	1.04 (0.92 to 1.18)	.64
12 mo	569 (25.2)	769 (25.6)	1.06 (0.93 to 1.20)	.49
**Secondary outcomes**				
Continuous daily prescriptions in past 28 d				
Baseline	499 (22.3)	671 (22.5)	NA	.60
3 mo	379 (16.8)	564 (18.9)	0.96 (0.71 to 1.31)	.84
6 mo	483 (21.5)	647 (22.0)	0.99 (0.73 to 1.35)	.63
9 mo	498 (22.0)	662 (21.9)	1.01 (0.74 to 1.37)	.56
12 mo	389 (17.3)	590 (19.6)	0.88 (0.65 to 1.22)	.66
Days on antipsychotic in the past 28 d, mean (SD)				
Baseline	6.76 (11.85)	6.96 (11.94)	NA	.39
3 mo	6.37 (11.50)	6.75 (11.77)	−0.29 (−0.73 to 0.15)	.96
6 mo	6.61 (11.75)	6.79 (11.84)	−0.16 (−0.67 to 0.34)	.72
9 mo	6.59 (11.74)	6.65 (11.77)	−0.01(−0.57 to 0.55)	.40
12 mo	6.54 (11.62)	6.47 (11.60)	0.04 (−0.56 to 0.63)	.32
Daily antipsychotic dose in the past 28 d, mean (SD)				
Baseline	115.45 (147.38)	118.09 (147.13)	NA	.90
3 mo	117.17 (134.80)	126.76 (155.92)	−4.22 (−11.65 to 3.20)	.62
6 mo	120.97 (147.56)	118.68 (146.80)	−1.24 (−10.19 to 7.70)	.81
9 mo	126.13 (151.67)	126.88 (152.16)	0.56 (−9.56 to 10.68)	.93
12 mo	127.22 (158.97)	124.21 (151.80)	0.68 (−11.20 to 12.57)	.32

Abbreviation: NA, not applicable.

^a^ Intervention effect for daily antipsychotic use in past 7 days (primary outcome) and continuous daily prescriptions in past 28 days (secondary outcome) presented as odds ratio with 95% CI; for mean days on antipsychotic in past 28 days and mean daily antipsychotic dose in past 28 days, mean difference with 95% CI.

Mean (SD) days on antipsychotics were similar between intervention and usual care at baseline (6.76 [11.85] days vs 6.96 [11.94] days; *P* = .39) and 12 months (6.54 [11.62] days vs 6.47 [11.60] days; *P* = .32), with no statistically significant change in the mean difference (0.04 days; 95% CI, −0.56 to 0.63 days). There were also no differences for the mean (SD) daily antipsychotic dose in the past 28 days between intervention and usual care at baseline (115.45 [147.38] mg vs 118.09 [147.13] mg; *P* = .90) and 12 months (127.22 [158.97] mg vs 124.21 [151.80] mg; *P* = .32), with no significant difference in the between-group change from baseline (mean difference, 0.68 mg; 95% CI, −11.20 to 12.57 mg).

The intervention group experienced a greater reduction at 6 months for mean (SD) clinical scores in pain (0.30 [0.59] vs 0.38 [0.66]; *P* < .001) and depression (2.18 [2.37] vs 2.81 [2.65]; *P* < .001) (Table 3). The proportion of patients receiving other medications did not appear to change in either group (eTable 3 in Supplement 2). Additional sensitivity analyses were undertaken, and their results are described in eTable 4 and eTable 5 in Supplement 2.

**Table 3.** Secondary Clinical Outcomes

Outcome	Participants, No. (%)		*P* value
	Intervention (n = 2303)	Control (n = 3060)	
Clinical events			
Nonelective hospitalization	405 (17.6)	551 (18.0)	.70
Unplanned ED visit	490 (21.3)	599 (19.6)	.13
Falls in last assessment	249 (10.8)	314 (10.3)	.77
Clinical scores, mean (SD)^a^			
ADL	15.43 (5.81)	15.08 (6.34)	.05
ABS	1.56 (2.25)	1.56 (2.12)	>.99
Pain	0.30 (0.59)	0.38 (0.66)	<.001
DRS	2.18 (2.37)	2.81 (2.65)	<.001

Abbreviations: ABS, Aggressive Behavior Scale; ADL, Activities of Daily Living; DRS, Depression Rating Scale; ED, emergency department.

^a^ Based on most recent quarterly assessment. ADL is scored 0 to 24; ABS, 0 to 12; pain, 0 to 3; and DRS, 0 to 14.

## Discussion

We performed a randomized clinical trial of academic detailing in partnership with health system administrators to reduce antipsychotic prescriptions in nursing homes. Homes receiving academic detailing did not achieve additional reduction in the prescribing of antipsychotics compared with standard quality improvement supports. The relative reductions in the proportion of residents receiving an antipsychotic prescription in the prior 28 days in the intervention and usual care groups were 22.4% and 12.8%, respectively, but this difference was not statistically significant. Of note, a simple before-and-after study design would have come to a completely different conclusion regarding the effectiveness of the intervention.

This study occurred during a time of system-wide reductions in antipsychotic prescribing in Ontario. Our embedded process evaluation,^21^ along with our time-series analysis, suggest that this overall reduction may be attributable in part to public and media reporting in the region.^34^ In addition, recent work has highlighted the effect of audit and feedback alone on producing a small but significant reduction in antipsychotic prescribing in nursing homes.^35,36^ Importantly, there was no observed risk in the use of other drugs (eTable 3 in Supplement 2); previous interventions have noted increased utilization of other medications as prescribers move away from monitored agents.^37,38^ Switching from 1 high-risk medication to another should be monitored as the downward trend of antipsychotic prescribing continues.

The statistically significant improvement in clinical scores related to pain and depression highlights important potential benefits of academic detailing in nursing homes. Importantly, the reductions in the scores border on those deemed clinically significant in previous studies but may not be conclusive of a clinically meaningful difference.^28,33,39^ These findings may reflect aspects of the intervention encouraging comprehensive assessment of behavioral and psychological symptoms of dementia and nonpharmacologic management as a first line of treatment, which were reported by intervention participants.^21^ In homes where efforts were likely already directed toward improving antipsychotic prescribing, the flexibility of an academic detailer to focus on related issues, such as management of pain and depression, may offer value.^21^ Participants in the embedded process evaluation further emphasized improved team functioning, including improved documentation and interprofessional communication, both important precursors for improving quality of care.^21,22^ Academic detailing is perceived as a credible, nonbiased external source,^21,40^ making it a promising strategy to productively influence both prescribing and related behaviors among health care professionals. The lack of clinically significant effects seen in this evaluation compared with other studies of detailing initiatives^17,41^ may reflect difficulty in making additional changes beyond those already initiated in the sector, the brief time that detailers had to develop influential relationships in the homes, the characteristics of the intervention, and/or the nature of those clinicians who engaged. Most evidence supporting the use of academic detailing takes place in settings where no other ongoing interventions or secular trends are concurrently occurring.

### Limitations

This study has limitations. It may be limited by the voluntary nature of enrollment at the home level. However, when the homes in the study were compared with the provincial average of antipsychotic prescribing, there was no significant difference. Furthermore, voluntary participation of staff and clinicians within homes led to considerable variation in the number of clinicians engaged across participating homes,^21^ which resulted in varying levels of exposure at both the home and clinician level. It is plausible that a threshold level of exposure required to achieve an effect was not consistently achieved (ie, inadequate intervention fidelity). Participants in the qualitative process evaluation reported that more comprehensive changes were observed when frontline clinicians, including personal support workers and nurses, were engaged in addition to physicians and pharmacists. Another potential limitation is the reliance on data that were not collected specifically for the study. This pragmatic choice provided a picture of real-world effectiveness to inform policy but makes it difficult to answer specific efficacy questions, including whether those who engaged in both the academic detailing and feedback reports achieved greater improvements in prescribing. This work was part of a larger assessment, presented elsewhere, that explored the feasibility and perceptions of this intervention.^21,22^ Furthermore, conclusions regarding the effectiveness of academic detailing based on a small number of initial encounters for a topic chosen by policy makers rather than by health professionals, may be premature, given the existing trend toward deprescribing and the likelihood that the intervention works better over time as relationships are developed.

## Conclusions

The findings of this study suggest that academic detailing in nursing homes may not be an effective intervention to reduce antipsychotic utilization in an environment where standard quality improvement supports are already creating desirable changes in prescribing. Our findings highlight the need for policy makers to carefully consider the topic and timing of such interventions.

## Notes

Supplement 1. **Trial Protocol**

Supplement 2. **eTable 1. **Description of Measured Variables and Data Source **eTable 2. **Description of Interactions **eTable 3. **Other Prescription Drugs Dispensed in Past 14 Days **eTable 4. **Discontinuation of Antipsychotic Among Residents Living in Nursing Homes at Baseline **eTable 5. **Summary of Intervention using TiDieR Suggested Categories

Supplement 3. **Data Sharing Statement**
