# Supplementary material for: Effect of Academic Detailing on Promoting Appropriate Prescribing of Antipsychotic Medication in Nursing Homes: A Cluster Randomized Clinical Trial
Source: JAMA Netw Open. 2020 May 26;3(5):e205724. doi: 10.1001/jamanetworkopen.2020.5724 (PMC7251442; doi:10.1001/jamanetworkopen.2020.5724)
Supplement: Supplement 1. — Trial Protocol [file jamanetwopen-3-e205724-s001.pdf]

APPROPRIATE PRESCRIBING DEMONSTRATION PROJECT: CLUSTER TRIAL PROTOCOL VERSION 3.0 DATE: 30 May 2016

Background and Rationale

As part of the 2012 Physicians Services Agreement, the Ministry of Health and Long-Term Care (ministry) and Ontario Medical Association (OMA) established an Appropriate Prescribing Work Group (APWG) tasked to make recommendations to the Physician Services Committee (PSC) on opportunities to improve prescribing in Ontario through voluntary quality-based educational strategies that respect the confidentiality of prescriber practice data. The PSC has approved an initial Demonstration Project of integrated educational strategies starting in Long-Term Care (LTC) homes. The stakeholders decided that appropriate antipsychotic prescribing would be the initial focus of the educational strategies in LTC. This was an appropriate choice as this drug class is known to have substantial risks in the elderly, to be relatively overprescribed given the known risk/benefit ratio, and to be amenable to quality improvement interventions in the LTC setting. The PSC subsequently decided to extend the educational outreach intervention to include falls as a second topic.

Antipsychotics: evidence for harms

Antipsychotic medications are typically prescribed in LTC facilities to manage the behavioural and psychological symptoms of dementia (BPSD). However, a significant association has been made between antipsychotic use and increased morbidity and mortality among elderly residents with dementia (Thompson Coon et al., 2014), signalling the pressing need for solutions regarding the appropriate treatment and management of symptoms. Residents are particularly at risk for inappropriate prescribing as they are more fragile, receive interventions from multiple point-of-care staff and are unable to detect errors in their medication. For this reason, residents of LTC facilities may receive up to four times as many prescriptions compared to the elderly in the community (Loganathan, Singh, Franklin, Bottle & Majeed, 2011). The effects of inappropriate prescribing on LTC residents' quality of life are complex: Inappropriate prescribing is often linked to polypharmacy and in turn, adverse drug reactions, causing adverse outcomes, such as increased hospital admissions, increased morbidity and impaired functionality (Beers, 1992; Garcia-Collarte et al., 2014). Specifically, antipsychotic medications are known to be associated with cardiovascular events, parkinsonisms, falls, and death (Rochon, 2007).

Antipsychotics: evidence of over-use

Ontario has one of the highest rates in Canada and is known to have one of the highest rates in the world of antipsychotic use. The Canadian Institute for Health Information (2014) reports that 30.5% of LTC residents are inappropriately prescribed antipsychotic medications, without a diagnosis of psychosis. According to a recent report from the Ontario Drug Policy Research Network, in 2012, 38.0% of residents in LTC were dispensed an antipsychotic.

Antipsychotics: predictors of over-use

Residents of larger nursing homes are more likely to be prescribed a greater number of inappropriate medications than those in smaller nursing homes (Beers et al., 1992, Ray et al., 1980). Kleijer and colleagues (2014) found that smaller, non-urban LTC facilities with high resident satisfaction report the lowest prevalence of antipsychotic drug use. Similarly, in a study by Hagen and colleagues (2005), it was determined that LTC residents in urban areas received more neuroleptics and benzodiazepines than their LTC counterparts in rural areas (26.1% vs. 15.7%) and 18.0% vs. 7.6% respectively).

Residents having more than one prescriber, having a physician aged 50 years of age or older, or having a non-specialist physician were more likely to be prescribed inappropriate antipsychotic medication (Dhalla et al., 2002). A qualitative analysis of the factors related to psychotropic drug prescription for neuropsychiatric symptoms in LTC residents with dementia revealed important themes (Smeets et al., 2014). Appropriate decision-making, such as "weighing the effectiveness of medications against its side effects and balancing the interests of different stakeholders", strongly contributed to the prescription of

psychotropic drugs. When providers had limited knowledge of antipsychotic risks and benefits, a greater need for psychotropic drug use among residents was cited. Effective communication and collaboration between professionals in LTC was found to deter or prevent the need for excessive prescription of psychotropic drugs. Lastly, limited personnel to spend enough time with residents, excess employment of temporary nurses, and the employment of nurses with limited people skills, was cited was increasing the need for psychotropic drugs (Smeets et al., 2014).

Antipsychotics: evidence for interventions to reduce prescribing in LTC

In a systematic review conducted by Thompson Coon and colleagues (2014) on interventions to reduce inappropriate prescribing of antipsychotics among people with dementia in LTC homes, a 12-20% reduction in antipsychotic prescribing levels was found. The type of intervention implemented has an effect on the size of improvements in initiatives targeting antipsychotic improvement in LTC homes. In a systematic review of interventions to optimize prescribing in LTC homes, academic detailing was found as having the strongest evidence for improvements in prescribing, whereas audit and feedback interventions provided small to moderate impact on physician practices (Loganathan et al., 2011). Furthermore, according to Milta and colleagues (2013), educational outreach interventions demonstrate improvements ranging from 18 to 19 percent reductions in antipsychotic use compared to geriatric assessment alone. It has been recommended that in order for educational interventions to be effective at improving inappropriate prescribing, they must be implemented alongside complementary techniques such as academic detailing (Loganathan et al., 2011; Milta et al., 2013). The development of a tracking mechanism that includes specific outcome measures is also recommended (Milta et al., 2013). A randomized control trial in which staff was randomly assigned to receive quarterly audit and feedback plus education about quality improvement demonstrated a decrease in resident falls, behavioural symptoms and pressure ulcers (Rantz et al., 2001).

Addressing the Risk of Falls in Long-Term Care

Falls are common occurrence among residents in LTC, with approximately 14.8% of Ontario LTC residents and 15.3% of Canadian LTC residents experiencing at least one fall within a 30 day period (CIHI, 2014). Approximately half of all LTC residents will fall at least once per year,

A previous fall triples the risk of future falls (Taylor et al, 2005). A plethora of risk factors have been identified, including age, physical conditions, medications, and behaviour, among others (Balash et al., 2005; Bloch et al., 2011; Dyks and Sadowski, 2015; Tinetti et al., 1992). The majority of these factors are modifiable, presenting an opportunity for the health care team to intervene. A systematic review of interventions to reduce falls in LTC concluded that multifactorial interventions hold more promise (Neyens et al., 2011), which aligns well with the strategy of educational outreach adopted by the APWG.

Objectives

This pragmatic, cluster randomized controlled trial seeks to assess the impact of an intervention that could be readily implemented at scale to improve appropriateness of prescribing. The policy-makers previously determined that all LTC prescribers in the province would be provided with the opportunity to access online practice reports (audit and feedback) that would confidentially describe their prescribing practices for antipsychotic medications, compared to (de-identified) peers. In addition, based on evidence suggesting the potential synergistic effects of audit and feedback with educational outreach (Ivers et al., 2012; Avery et al., 2012), policy makers decided that an educational outreach should be included as part of an 'Appropriate Prescribing Demonstration Project'.

This proposal forms one part of a program evaluation for the Demonstration Project; given the costs inherent in delivering educational outreach, a rigorous design to assess the added impact of this intervention is desirable. A mixed methods formative evaluation and process evaluation are planned to understand how and why program effects were observed and provide insights regarding scale-up of the interventions.

Primary question:
What is the effect of adding educational outreach (AF+EO) compared to the 'usual' offering of audit and feedback (AF) and communities of practices on:

1. The prescribing of antipsychotic medications in LTC
2. The rate of falls in LTC

Secondary questions:
What is the impact of the interventions on clinical outcomes?

i. What is the effect of the intervention on acute care utilization (e.g., emergency room) rates?
ii. What is the effect of the intervention on incidence of patient clinical outcomes and/or adverse effects associated with APMs (e.g., falls, aggressive behaviours)?
iii. What is the effect of the interventions on medications that might be used as alternatives to APMs (e.g., benzodiazepines)?
iv. What is the effect of the intervention on medications that increase the risk of falls among residents of long-term care (e.g. benzodiazepines, antihypertensives, opioids, anticholinergics)?

What are the economic implications of the intervention?

v. What is the cost-benefit when focusing only on prescribing outcomes?
vi. What is the cost-effectiveness considering all MOHLTC-relevant outcomes?

How and why do the interventions work as observed?

vi. Was the intervention implemented as desired in LTC homes?
vii. What were the contextual factors associated with implementation?
viii. Do the interventions affect precursors of behaviour (e.g., motivation, capability)?

<u>Design</u>

Two arm, pragmatic, cluster-randomized trial, with LTC homes allocated to the full, active intervention (featuring educational outreach offered to each prescriber and team members in the home) or standard quality improvement supports (including online audit and feedback reports for each prescriber in the home). The 'standard' quality improvement supports represent 'usual care' as these are to be launched province-wide; a concurrent control arm with no exposure to a quality improvement intervention is not feasible.
[ClinicalTrials.gov NLM Identifier: NCT02604056]

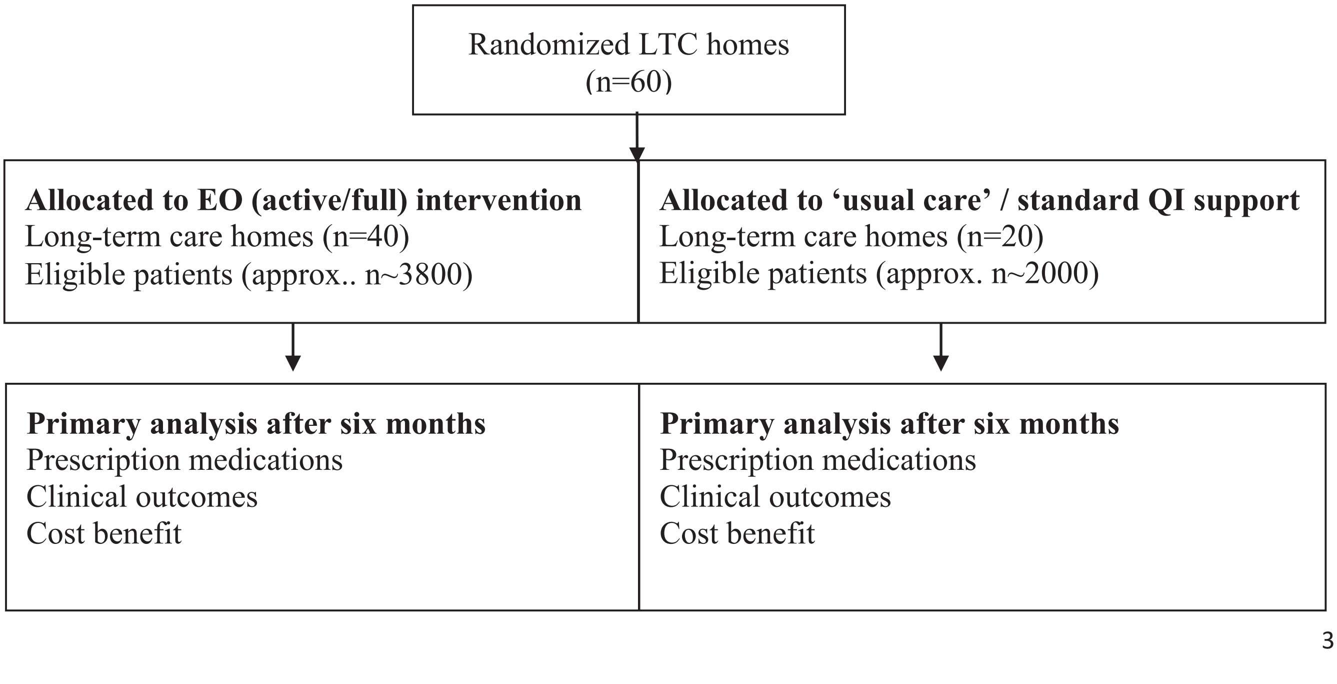

To evaluate the secondary aim of how and why the interventions work as observed, we will employ an embedded single case design with cross case synthesis to gain an in-depth understanding of the conditions and factors associated with implementation of the intervention. The embedded design enables the examination of interactive processes and contextual features through various units of analysis derived from a structured approach to develop programme theory (Davidoff et al., 2015; Portela et al., 2015; Reed et al., 2014). Normalization Process Theory (NPT) and the Consolidated Framework for Implementation Research (CFIR) will be used to guide and structure data collection methods and data analysis. NPT is an established framework for understanding how and whether complex interventions become embedded in health care practice—that is, normalized (Macfarlane et al., 2012). This approach is ideally suited to this study as it entails numerous individuals, professionals, unit, organizational and health systems features that may impact the effectiveness of the audit and feedback intervention in selected LTCs. The CFIR provides a comprehensive taxonomy of defined constructs that are likely to influence implementation (Damschroder et al., 2009). Its application facilitates the identification of active components and further explores the setting in which the intervention is delivered.

Eligibility

LTC homes within pre-determined regions of Ontario are eligible if they express an interest in the full intervention. These regions, or hubs, were chosen because they contain a wide variety of LTC home types within a reasonable travel distance (i.e., <100 km). To be eligible for participation, the medical and administrative leads of the LTC homes must agree to and support the project. Exclusion criteria include: LTC homes with a previous or ongoing involvement in externally supported quality improvement initiatives focusing on antipsychotics; LTC homes without any prescribers caring for at least 10 residents routinely; or LTC homes with fewer than 30 residents. For the topic of antipsychotic medication, we will conduct qualitative interviews in a subset of 3-4 LTC homes that represent the Hamilton geographical hub. The sampling strategies will consist of three variants of purposeful sampling: maximum variation, convenience and snowballing sampling technique. Maximum variation will be used when identifying the LTC homes. The selected homes will represent variations of antipsychotic prescribing rates from high to low. For the topic of falls reduction, we will conduct qualitative interviews in a subset of 5-6 LTC homes that represent the variation that exists within the study sample (e.g. number of beds, urban vs. rural, type of ownership). The sampling strategies will consist of three variants of purposeful sampling: maximum variation, convenience and snowballing sampling technique. Maximum variation will be used when identifying the LTC homes. The selected homes will represent variations among the rate of falls from high to low.

Allocation

Participating LTC homes will be allocated to one of the two arms of the study (active/full intervention versus standard education) using a 2:1 allocation ratio, after stratifying on hub, baseline antipsychotic prescription rates (using published CIHI reports), and size (number of eligible patients). A 2:1 allocation ratio will be used to satisfy the requirements of the sponsor and to improve feasibility of recruitment of homes. LTC homes that share prescribers will be allocated as a unit to avoid contamination. Due to funding restrictions, no more than 38 homes will be allocated to the intensive intervention; if more than 60 homes are recruited, the allocation ration will be less than 2:1, but the odds of allocation to the full intervention will remain greater than 1:1. Allocation will be conducted by an independent statistician using computer-generated random numbers.

Interventions

Standard quality improvement supports / Usual care:
Numerous quality improvement initiatives are simultaneously occurring in the LTC sector related to appropriate prescribing, making a randomized comparator all the more important. For instance, CIHI is reporting on antipsychotic use and antipsychotic prescribing has been named a ‘priority indicator’ for mandatory annual quality improvement plans to be completed by each LTC.
Starting in autumn 2015, in concert with the initiative outlined in this proposal, HQO will be producing confidential **practice reports** using data from ICES to describe selected quality indicators for LTC physicians. This will initially focus on appropriate prescribing of antipsychotics for older men and women with dementia, with an initial focus on how their antipsychotic medication use compares to others in the province plus additional information regarding the clinical and demographic features of their LTC patient roster. Receipt of the reports is voluntary. To receive the reports, physicians must sign up and confirm their identity. They will then receive updated reports quarterly, which they can download from HQO’s secure website. In addition, HQO will be launching voluntary, virtual ‘communities of practice’ to enable sharing of best practices across homes and providers. Starting in autumn 2016, HQO will introduce additional indicators to the practice reports, including benzodiazepines, antihypertensives, and anticholinergic medications.
Active intervention:
The **educational outreach** program will be delivered by academic detailers, health professionals (often nurses or pharmacists) who have received specific intensive training to support prescribing providers in a particular environment, in this case LTC homes, to make the best or appropriate clinical decisions as possible. It is defined as: "*an innovative method of service-oriented educational outreach for front-line clinicians. It combines the interactive, one-on-one communication approach of industry detailers with the evidence-based, noncommercial information of academia.”* (http://www.narcad.org/about/aboutad/)
The intervention requires the Academic Detailers to have direct and ongoing contact with the LTC homes including administrators, providers and staff from the time of launch through to program completion. Given the diverse target audiences that will likely be engaged within the homes, the Academic Detailer will work to understand the context, barriers, capacity, and needs of each home to ensure the service being provided by the detailer is relevant, meaningful and appropriate. The intervention then includes the provision of the following different 'types' of visits made by the Academic Detailer: meetings (likely with administrators to describe the program and value), presentation (large group, inter professional audience), group visit (typically 2-6 providers) and one-on-one visits ('traditional' Academic Detailing visits). In addition, the Academic Detailer provides important service to the LTC homes and providers between visits (i.e., by responding to questions via email or phone) to respond to the needs and build trust/credibility over time.
The Centre for Effective Practice, a Toronto-based non-profit organization retained by the OMA/MOHLTC due to their extensive experience in the area, will manage the educational outreach intervention. Best practices in training of the Academic Detailers and in the components of the program will be applied. Given the nature of the program as a Demonstration Project for the purposes of understanding feasibility in the Ontario context, it is critical to ensure a pragmatic approach to the delivery of the intervention. Therefore, the number of visits per provider or home per topic and the length of visits will not be pre-defined.

Data collection

Outcomes will be assessed using population-level administrative databases linked through unique, encrypted patient identifiers at the Institute for Clinical Evaluative Sciences (ICES). Data will be compiled from the following databases: 1) the Ontario Drug Benefits (ODB) database, covering all prescription medications dispensed to residents in long-term care homes; 2) the Canadian Institute for Health Information (CIHI) databases covering all hospitalizations and emergency department visits; 3) the Ontario Health Insurance Plan (OHIP) database, covering physician billings for procedures and

consultations; 4) the Registered Persons Database covering demographic information including date of death; and 5) the Continuing Care Reporting System (CCRS) database for clinical and demographic information on LTC residents collected using the Resident Assessment Instrument (RAI). Each LTC resident in the province receives a 'full' RAI assessment within 2 weeks from admission date, every 12 months from previous full assessment or any other time when the clinical condition of a patient changed considerably and each resident also receives a 'quarterly' assessment every 3 months. For each resident, the CCRS database holds demographic and clinical information, including data regarding clinical diagnoses (e.g., dementia, psychosis), dispensing of antipsychotic medications, and occurrences of falls. In addition, a number of validated outcome scales can be derived from the CCRS data, including those related to ADLs, aggressive behaviour, pain, and mood.

The process evaluation involves a mixed-methods approach using individual qualitative interviews and quantitative prescriber surveys.

LTC Administrators and Prescribers

We will seek interviews with home administrators, medication prescribers including physicians and nurse practitioners, and consultant pharmacists. A research coordinator will inform participants of the nature of the study being to understand how stakeholders respond to and interact with the interventions and also to explore for any unintended consequences—exploring their experiences associated with the implementation of the academic detailing intervention.

Academic Detailers

All academic detailers will be invited to participate in interviews. A research coordinator will inform the academic detailers of the nature of the study - exploring their experiences associated with the implementation of the academic detailing intervention.

Interview Guides

Written consent will be obtained prior to the interviews (see attached Interview Consent Forms). The interviews will be guided by the detailed program theory (which itself will be refined iteratively), the CFIR, and NPT (Damscroder et al., 2009; MacFarlane et al., 2012; Portela et al, 2015) (see Administrator & HCP Interview Guide). The interview guides will be pilot tested prior to their full-scale use. Participants will be informed that participation in the interview of the study is voluntary and they can withdraw from the study at any time without penalty to their employment. It is expected interviews will last between 30 to 60 minute interviews. Participants will also be informed that they can skip questions during the interview without affecting their participation in the study and that interviews will be digitally audio taped and transcribed verbatim.

Prescriber Survey

To understand the intervention's mechanism of change, we will survey participating clinicians and LTC administrators in both groups prior to intervention delivery and again 6 months later to measure theoretical constructs targeted by our intervention and changes in these constructs associated with the intervention (Grimshaw et al., 2007). We will assess constructs based on the COM-B model (capability, opportunity, and motivation) (Michie et al., 2011), which are specifically targeted by the intervention. These constructs will be assessed for each of the targeted clinical behaviours. Questions will include validated items from the Determinants of Implementation Behaviour Questionnaire (Huijg et al., 2014) as well as measurements of self-efficacy and coping planning, two factors which have been shown to influence clinician behaviour (Presseau et al., 2014).

Outcomes

The following primary and secondary prescribing and clinical outcomes will be assessed in the trial:

Variable	Definition	Source
**Primary outcome**		

Antipsychotic dispensing	Number of days with antipsychotic prescriptions in the last week (count, range 0 – 7)	RAI
**Secondary prescribing outcomes**		
Antipsychotic prescribing	Any antipsychotic Rx during the past month (dichotomous)	ODB
Mean Antipsychotic dose	Dose equivalent of antipsychotic dispensed in the past month (continuous)	ODB
Benzodiazepine (or z-drug) prescribing	Any Rx during the past month (dichotomous)	ODB
Anti-depressant prescribing	Any Rx during the past month (dichotomous)	ODB
Acetaminophen prescribing	Any Rx during the past month (dichotomous)	ODB
**Secondary clinical outcomes**		
Difficulty in performing activities	ADL long form scale (continuous variable, range 0-28)	RAI (ADL_long_cc)
Aggressive behaviour scale	Extent of aggressive behaviour (continuous variable, range 0-12)	RAI (ABS_cc)
Pain	Pain scale (continuous variable, range 0-3)	RAI (PAIN_cc)
Depression	Depression rating scale (continuous variable, range 0-14)	RAI (DRS_cc)
Falls	Number of falls in the past month (count)	RAI (J4: a)
**Secondary health care utilization outcomes**		
ER visits	Number of ER visits during the previous 6 months (count)	CIHI/NACRS
Hospitalizations	Number of hospital visits visit during the previous 6 months (count)	CIHI

All **primary and secondary prescribing outcomes** will be assessed at baseline (pre-intervention) and at 3 and 6 months post-intervention. Here, "baseline" is defined as the week or month immediately prior to randomization, while post-intervention measurements are defined as the third and sixth months after randomization. These outcomes will be assessed on all residents who are alive at the time of the assessment and who were present in the home for the full duration of the prior week/month.
All **clinical outcomes** (e.g., ADL, behaviour scale, depression, falls) will be assessed at baseline and at 3 and 6 months post-intervention. The most current data collected immediately prior to the implementation of the intervention, and at each post-randomization time point will be used, regardless of mortality status at the subsequent time point.
All **health care utilization outcomes** (number of ER visits and hospitalizations during the previous 6 months) will be assessed at baseline and at 6 months post-intervention. A longer duration is required to allow adequate time for the intervention to have an effect on these outcomes. All residents present in the homes at any time during the study will contribute to the analysis using person-time of follow-up.
As the EO intervention may be rolled out over a 2-3 month period due to logistical restraints, homes allocated to the control arm will have their measurement times assigned randomly from the assessment times of the intervention homes in the same stratum.

Analysis

Descriptive statistics will be calculated for all variables of interest: continuous variables with a normal distribution will be described using means and standard deviations (medians and inter-quartile ranges in the case of skewed distributions), whereas categorical variables will be summarized using frequencies and proportions. All analyses will be by Intention To Treat. Analyses will be conducted using SAS v.9.3 and statistical significance will be assessed at the 5% level.
The **primary outcome** describing **dispensing of medication** (number of days with antipsychotic/benzodiazepine/antihypertensive/anticholinergic prescriptions in the last week) will be analyzed using generalized linear mixed effects regression with multinomial distribution and cumulative logit link. To account for the staggered implementation of the intervention, phase (phase1/phase2) will be included as a fixed effect. Random intercepts and slopes will be specified to account for intracluster

and inter-period correlations. To maximize statistical efficiency, the analysis of prescribing outcomes will adjust for the stratification factors as well as public/private home ownership and the following patient covariates: age, sex, levels of function and aggressive behaviour (using multiple imputation for missing covariates if necessary). The effect of the intervention at 6 months will be expressed as cumulative Odds Ratio (OR) with 95% confidence interval (CI). In case of non-convergence or violation of the proportional odds assumption using the score test, the normal distribution and identity link will be used. The dichotomous **prescribing** outcomes (i.e., any relevant prescription in the last month) at baseline, 3 months, and 6 months will be analyzed using generalized linear mixed effects regression with binomial distribution and logit link function. Fixed and random effects will be specified as described for the primary outcome. The fixed effects of time, intervention, and intervention by time will be the main variables of interest. Dose equivalent of antipsychotic dispensed in the past month at baseline, 3 months, and 6 months will be analyzed using linear mixed effects regression with normal distribution and identity link. Fixed and random effects will be specified as described for the primary outcome. The effect of the intervention will be described as difference in change from baseline to 6 months using adjusted least square means with 95% CI. In the case of substantial skewness, gamma distribution with log link or log-normal distribution will be considered, and difference in change from baseline will be expressed as adjusted relative mean difference with 95% CI.

Secondary clinical outcomes measured as continuous variables (e.g., ADL, depression score) will be analyzed using linear mixed effects regression with normal distribution and identity link. Fixed and random effects will be specified as described for the prescribing outcomes. The effect of the intervention will be expressed as adjusted least square mean differences with 95% CI. Secondary clinical outcomes measured on an ordinal scale (e.g., pain scale) at baseline, 3 months and 6 months will be analyzed using generalized mixed effects regression as described for the primary outcome but using multinomial distribution and cumulative logit link. Presence of any falls in the past 30 days will be analyzed at baseline, 3 months, and 6 months using generalized linear mixed effects regression with binomial distribution and logit link, as described for the primary outcome. In the Intent-To-Treat analyses for the secondary clinical outcomes (e.g., ADL, falls, pain), the potential effect on inferences of patients who died will be examined using sensitivity analyses under conservative assumptions: for example, patients who died during the interval will be assigned the worst possible score. Depending on the extent of patient turnover within homes, additional analyses for primary and secondary outcomes will explore inclusion of subject-specific random effects to account for repeated measures on the same patient over time (i.e., a cohort rather than cross-sectional design).

Health care utilization outcomes (number of ER visits and hospital visits) will be analyzed at 6 months using generalized linear mixed-effects regression with Poisson or negative binomial distribution and log-link, with log person-time of follow-up as an offset term. The following fixed effects will be included: intervention, phase, and stratification variables, as well as the rate of health care utilization at the home 6 months prior to the intervention. Patient covariates will be adjusted for as specified for the primary outcome. Home will be included as a random effect. The effect of the intervention will be expressed as adjusted Rate Ratio (RR) with 95% CI.

Potential effect modification by ownership status of LTC home and staffing ratios, size of the home (<65 beds, 65-129 beds, or >129 beds), location (urban/rural), specialist utilization (proportion of patients with geriatrician or psychiatrist consultation within 6 months), plus primary physician characteristics (sex, years' experience), as well as patient characteristics (age, sex, time at facility, levels of function and aggressive behaviour plus whether a geriatrician or psychiatrist provided a consult) will be explored by including interactions between these variables and: time, treatment, and treatment by time.

Although primary analyses will examine outcomes for all patients excluding those with known to be palliative or with a history of bipolar disorder, schizophrenia or Huntington's disease, planned sub-group analyses will focus on those with dementia. A planned sensitivity analysis will restrict the educational

outreach cohort to physicians who also accessed the audit and feedback to examine for synergy in the full intervention group. We do not anticipate substantial missingness on prescribing or health care utilization outcome; however, exploratory analyses will be carried out to identify the extent of missing data on resident clinical and demographic variables (e.g., levels of function, aggressive behaviour, depression). If more than 5% of residents have missing data at any time, we will create a complete data set for analysis using multiple imputation, where the imputation model will include all covariates of interest in subsequent primary and secondary outcome analyses, as well as those variables identified as associated with missingness.

Since the CCRS data is only released for linkage at ICES annually, a planned interim analysis will compare the primary and secondary prescribing outcomes (which use ODB data). No stopping rules are planned based on this interim analysis; the results will be considered tentative and used only for confidential reporting to the funders.

Qualitative data will be analyzed using Charmaz's textual analysis method (Charmaz, 2014). NPT and the CFIR, used to inform the development of the interview guides, will be used to guide coding and analysis of the qualitative data (Damscroder et al., 2009; MacFarlane et al., 2012). In the final analytical phase, the investigative team and research personnel including trainees will prepare a comprehensive case study database with the datasets from each of the research questions (Yin, 2009). Enhancing credibility of case study design involves the use of multiple data sources and methods (Yin, 2009). We will triangulate key themes, categories and codes comparing and contrasting the case study database with the evolving programme theory hypotheses (cross-syntheses). Through a series of 2-3 analytical sessions, the case report will be finalized. We will employ a variety of strategies to ensure fidelity and credibility of the data: 1) using multiple sources of data, writing a chain of evidence that describes all elements of the case study database and a description of the derivation of the evidence from initial research questions to ultimate case study conclusions, and having key informants review the case study draft by having collaborators participate in the triangulation analysis and the return of findings (construct and external validity); 2) examining points of convergence (pattern matching) and divergence (examining alternative explanations) within and amongst the various datasets (internal validity through cross comparative analyses); and 3) creating a case study database with a chain of evidence, and having a stepped analysis process whereby there is an initial independent review of the data (transcripts, field notes) by at least 3 reviewers who then meet to reach consensus around the common themes (reliability) (Kidder and Judd, 1986; Lincoln and Guba, 1985).

We will analyse process evaluation data from clinician surveys after the intervention has been completed. Baseline data evaluation will include descriptive statistics, psychometric properties, and exploration of the association between process variables as a preliminary test of the theorised process models. This will be conducted by a statistician without knowledge of group allocation. Measures of capability, opportunity, and motivation will be calculated as the mean of the measure item scores.

We will use analytical methods previously developed to test for differences between groups on hypothesised targeted constructs, controlling for baseline differences (Grimshaw et al., 2007). Mediation models will be used to test whether intervention effects on behaviour are mediated through the targeted theoretical constructs.

Power

Our sample size is determined primarily by pragmatic considerations and logistical constraints. A total of **58 LTC homes** will be included in the trial: a maximum of 38 homes allocated to receiving the full intervention and 20 homes to receiving usual care. After grouping homes sharing providers together, we anticipate a total of 45 independent clusters (30 intervention, 15 control). For our primary outcome measured as an **ordinal** variable (number of days with antipsychotic prescription in the past 7 days), 45

homes with 2:1 allocation will yield 80% power to detect a cumulative Odds Ratio (i.e., odds of being in a higher category) of 0.6, assuming an intracluster correlation coefficient (ICC) of 0.01 and an average of 120 residents per home. Assuming that 30% of control arm patients will have 7 days of antipsychotic prescriptions, an OR of 0.6 implies a reduction in this proportion in the intervention arm to 20%.

To ensure we will be able to detect clinically important differences with adequate power for our secondary prescribing outcome (measured as a **dichotomous** variable), we used a simulation study with 1000 simulation runs to determine the required effect size (difference in change from baseline to 6 months in antipsychotic prescribing between the arms) that can be detected with at least 80% power. For this simulation, we assumed a conservative ICC of 0.1, an average of 120 residents per home, and a control arm proportion of 30%. With 45 homes (30 intervention, 15 control), we will have **83.4**% power to detect an OR of approximately 0.75 at 6 months. Assuming a control arm proportion of 30%, this OR corresponds to a reduction in the intervention arm to approximately 25%.

<u>Recruitment</u>

The recruitment process of LTC homes is described below.

In summary, recruitment of LTC homes will occur by emails co-signed by the OMA, MOHLTC, and local LHIN leads to eligible LTC home administrative leads and from other stakeholders (i.e., local physician leads) to medical directors of eligible LTC homes, with follow up phone calls as required. Some LTC homes have already expressed interest as they heard about the project being developed. These will be contacted again to confirm willingness to be involved in the interventions and the evaluation. Messaging during recruitment will focus on a few key concepts to mitigate concerns: 1) there is no downside, since randomization to not receive the outreach means continuing to function as usual with no added mandatory burdens or costs; 2) randomization will be skewed so that chances of receiving the intervention are greater than 50%; 3) we wish to observe what would happen in real-life, so consenting to the trial does not commit them to do anything they feel is not their residents' best interest; 4) evaluations will be conducted at the aggregate level to protect confidentiality.

To date, the project team have developed a plan that has identified >30 potential LTC homes in each region of the province where the project will occur. Recruitment will attempt to preferentially focus on LTC homes known to have higher antipsychotic rates. Identification of additional homes will be done in collaboration with the LHINs and other stakeholders and will be focused on first recruiting those homes with highest baselines antipsychotic prescribing rates and the lowest prescriber overlap. If greater than 58 homes are recruited, then the excess homes allocated will receive 'audit and feedback only' as the educational outreach will be limited to 38 homes total.

No recruitment of patients is required or planned.

Qualitative Interviews:

Initial contact will be from the investigative team (Drs. Noah Ivers and Dr. Laura Desveaux) with the LTC facilities' Senior Management and/or Director of Care/Nursing for the homes selected for the process evaluation. We will seek interviews with medication prescribers including physicians and nurse practitioners, consultant pharmacists, and administrators at each of the targeted homes. A research coordinator will inform participants of the nature of the study being to understand how stakeholders respond to and interact with the interventions and also to explore for any unintended consequences—exploring their experiences associated with the implementation of the academic detailing intervention. As a first wave of recruitment for health professionals in the embedded process evaluation, the leadership team of each home will be asked to distribute via email a letter of information (see Study Information Sheets) about this aspect of the larger study to the prescribing health professionals in the facilities where the case studies will be focused (December 2015-January 2016 and November-December 2016). The

email message will have pertinent information of the nature of the study. As a second wave of recruitment, if response is less than 25%, the email will be re-sent as a reminder to the health professional sample pool. The third and final wave of recruitment will consist of purposive sampling of prescribers and pharmacists associated with the home, using snowball techniques to seek providers with various perspectives. This method would entail existing participants to identify potential participants among their known acquaintances. All academic detailers will be invited to participate in an interview.

Prescriber Survey:
The survey will be administered by the evaluation team to the LTC home leadership. At the time of study launch and after 6 months, the evaluation team will distribute the survey, asking home leadership to complete it themselves in addition to forwarding the survey to the prescribers and pharmacists. The evaluation team will also send a series of weekly reminders to home leadership, for a total of three weeks, to encourage completion.

Ethical considerations: confidentiality and consent

There are no known risks associated with participation for LTC homes, the professionals working there, or the residents living there. Clinical decisions not dictated by the interventions. The quality improvement interventions being tested may each be considered variants of 'usual care' and could be implemented without any research ethics oversight if no evaluation was planned.

Participation is entirely voluntary. Leadership of the LTC homes (i.e., medical director and administrative leads) will be provided with time to review the relevant information prior to consent. Providers in the home can decide whether and when to engage with the actual educational interventions.

Outcome evaluations will be conducted using de-identified data available from the Institute for Clinical Evaluative Sciences (ICES). All evaluations will be reported at the aggregate level and the confidentiality of LTC homes, patients, and providers will be protected. The list of participants will be kept in a secure location.

Based on preliminary discussions with the research ethics experts, the entire home is considered a research participant and so explicit, written consent would be sought prior to allocation from the medical director and administrative leads from each home. A letter of information would be delivered post-allocation to all health care professionals in the LTC home. Waiver of consent for patients would be sought as the intervention does not directly impose treatment decisions or involve any direct interactions with residents. This **meets TCPS-2 requirements for waiver of consent** given minimal risk involved in providing education to health care providers. Of note, random allocation does not require consent of all participants; pursuing consent of all LTC prescribers working in the potentially eligible homes prior to allocation would be impracticable.

Individual consent will be obtained for participants volunteering to complete interviews. No subject identifiers of participants will be included in the conduct, data storage, analysis and presentation of qualitative findings (data will be presented in aggregate form only). To ensure confidentiality, all identifying features will be removed to protect participant anonymity. This is described in the information sheet to participants. Participation in the electronic survey will indicate implied consent. Only the Investigative Team under the supervision of the Principal Investigators will have access to the data prior to destruction. All electronic copies of data will be password protected.

Economic evaluation

Costing analyses

We will work with the teams conducting the intervention to determine the costs for start-up and implementation. Average costs will be presented as $/LTC home, $/LTC physician, and $/LTC patient.

Cost benefit and cost effectiveness analyses

A simplistic cost benefit report will be produced comparing costs of the intervention with direct savings related to changes in prescribing (if any) as well as the cost per type of clinical outcome achieved (e.g., $/Rx change). We will also conduct a cost effectiveness analysis from the perspective of the government. Costs associated with delivering (+/- designing) the intervention, along with health care utilization by patients will be compared. We will estimate the incremental cost per unit change in targeted behaviour (i.e., inappropriate prescribing) as well as the incremental net benefit of the intervention using mixed-effects regression analyses. Uncertainty will be addressed by estimating 95% CIs using a non-parametric bootstrapping method. A series of sensitivity analyses will be undertaken to examine underlying model assumptions.

<u>Implementation</u>

The audit and feedback will be launched and accessible for prescribers working in LTC in Ontario in September 2015. To accommodate the budgetary implications of training the personnel needed to conduct the educational outreach, the trial will rollout in ***two phases***, as described in the figure below:

	**JUL-AUG**	**SEPT-OCT**	**NOV-DEC**	**JAN 16 - FEB**	**MAR - APR**	**MAY-JUN**	**JULY-AUG**	**SEPT-OCT**	**NOV-DEC**	**JAN 17 - FEB**	**MAR**
CONSENT	X	AF open to all									
**Topic 1 (Antipsychotics)**											
20 homes ***PHASE 1***		Allocated to **EO (Topic 1)**		**Qualitative process evaluation**	6M follow up complete		6M ICES ODB data complete				
20 + homes ***PHASE 1***		Allocated to **AF only**		**Qualitative process evaluation**	6M follow up complete		6M ICES ODB data complete				**Potential launch of EO** (if scale-up approved)
20 homes ***PHASE 2***					Allocated to **EO (Topic 1)**			6M follow up complete		6M ICES ODB data complete	
**Topic 2 (Falls)**											
All Intervention Homes (n=40)						Introduction of EO Topic 2			**Qualitative process evaluation**		

References

Avery AJ, Rodgers S, Cantrill JA, Armstrong S, Cresswell K, Eden M, Elliott RA, Howard R, Kendrick D, Morris CJ, Prescott RJ, Swanwick G, Franklin M, Putman K, Boyd M, Sheikh A. A pharmacist-led information technology intervention for medication errors (PINCER): a multicentre, cluster randomised, controlled trial and cost-effectiveness analysis Lancet. 2012 Apr 7;379(9823):1310-9. doi: 10.1016/S0140-6736(11)61817-5.

Beers, M.H., Ouslander, J.G., Fingold, S.F., Morgenstern, H., Reuben, D.B. ... Beck, J.C. (1992). Inappropriate Medication Prescribing in Skilled-Nursing Facilities. *Medicine and Public Issues, 117,* 84-89.

Balash Y, Peretz C, Leibovich G, Herman T, Hausdorff HM, Giladi N. Falls in outpatients with Parkinson's disease: frequency, impact and identifying factors. J Neurol 2005; 252(11):1310–5.

Bloch F, Thibaud M, Dugue B, Breque C, Rigaud AS, Kemoun G. Psychotropic drugs and falls in the elderly people: updated literature review and meta-analysis. J Aging and Health 2011; 23(2):329–46.

Canadian Institute for Health Information. (2013). *When a Nursing Home Is Home: How Do Canadian Nursing Homes Measure Up on Quality?* Ottawa, ON: CIHI.

Canadian Institute for Health Information (2014). *Your Health System. [accessed May 27 2016]. Available at: http://yourhealthsystem.cihi.ca/*

Charmaz, K.(2014). Constructing grounded theory. 2nd ed. Sage.

Coon, J.T., Abbot, R., Rogers, M., Whear, R., Pearson, S., ... Stein, K. (2013). Interventions to Reduce Inappropriate Prescribing of Antispychotic Medications in People with Dementia Resident in Care Homes: A systematic review. *Journal of the American Medical Directors Association, 15*(10), 706-718.

Damschroder, L.J., Aron, D.C., Keith, R.E., Kirsh, S.R., Alexander, J.A., Lowery, J.C. (2009). Fostering implementation of health services research findings into practice: a consolidated framework for advancing implementation science. *Implementation Science, 4*(1), 50.

Davidoff, F., Dixon-Woods, M, Leviton, L, and Michie, S (2015). *Demystifying theory and its use in improvement. BMJ quality & safety. 24*(3),228-38.

Dhalla, I.A., Anderson, G.M., Mamdani, M.M., Bronskill, S.E., Sykora, K., Rochon, P.A. (2002). Inappropriate Prescribing Before and After Nursing Home Admission. *Journal of American Geriatrics Society, 50*(6), 995-1000.

Garcia-Gollarte, F., Baloeriola-Julvez, J., Ferrero-Lopez, I., Cuenilas-Diaz, A., Cruz-Jentoft, A.J. (2014). An Educational Intervention on Drug Use in Nursing Homes Improves Health Outcomes, Resource Utilization, and reduces Inappropriate Drug Prescription. *Journal of American Medical Directors Association,15,* 885-891.

Grimshaw,J.M., Zwarenstein, M., Tetroe, J.M., Godin, G., Graham,I.D., … Presseau, J. (2007). Looking inside the black box: a theory-based process evaluation alongside a randomised controlled trial of printed educational materials (the Ontario printed educational message, OPEM) to improve referral and prescribing practices in primary care in Ontario, Canada. *Implementation Science, 2*, 38.

Hagen, B.F., Armstrong-Esther, C., Quail, P., Williams, R.J., Norton, P., … Zieb, R. (2005). Neuroleptic and benzodiazepine use in long-term care in urban and rural Alberta: characteristics and results of an education intervention to ensure appropriate use. *International Psychogeriatrics, 17*(4), 631-652

Huijg, J.M., Gebhardt, W.A., Dusseldorp, E., Verheijden, M.W., van der Zouwe, N., … Crone, M.R. (2014). Measuring determinants of implementation behavior: psychometric properties of a questionnaire based on the theoretical domains framework. *Implementation Science, 19*(9), 33.

Ivers N, Jamtvedt G, Flottorp S, Young JM, Odgaard-Jensen J, French SD, O'BrienMA, JohansenM, Grimshaw J, Oxman AD. Audit and feedback: effects on professional practice and healthcare outcomes. Cochrane Database of Systematic Reviews 2012, Issue 6. Art. No.: CD000259. DOI: 10.1002/14651858.CD000259.pub3.

Kidder, L.H., and Judd, C.M. (1986). *Research Methods in Social Relations.* New York, NY: Holt, Rinehart & Winston.

Kleijer, B.C., van Marum, R.J., Frijters, D.H.M., Jansen, P.A.F., … Heerdink, E.R. (2014). Variability between nursing homes in prevalence of antipsychotic use in patients with dementia. *International Psychogeriatrics, 26*(3), 363-371.

Lincoln, Y.S., and Guba, E.G. (1985). *Naturalistic inquiry.* Vol. 75. Newbury Park, CA: Sage.

Loganathan, M., Singh, S., Franklin, B.D., Bottle, A., Majeed, A. (2011). Systematic Review: Interventions to optimize prescribing in care homes. *Age and Aging,* 1-13.

MacFarlane, A., and O'Reilly-de Brún, M. (2012). Using a theory-driven conceptual framework in qualitative health research. *Qualitative Health Research, 22*(5), 607-618.
Michie, S., van Stralen, M.M., West, R. (2011). The behaviour change wheel: a new method for characterising and designing behaviour change interventions. *Implementation Science, 6*, 42

Milta, O., Little, D.O., & Morley, A. (2013). Reducing Polypharmacy: Evidence from a Simple Quality Improvement Initiative. *JAMDA,* 152-156.

Neyens JC, van Haastregt JC, Dijcks BP, Martens M, van den Heuvel WJ, de Witte LP et al. Effectiveness and implementation aspects of interventions for preventing falls in elderly people in long-term care facilities: a systematic review of RCTs. J Am Med Dir Assoc 2011; 12(6):410–25.

Portela, M.C., Pronovost, P.J., Woodcock, T., Carter, P., Dixon-Woods, M. (2015). How to study improvement interventions: a brief overview of possible study types. *BMJ quality & safety*, *24*(5), 325-336.

Presseau, J., Johnston, M., Francis, J.J., Hrisos, S., Stamp, E., … Eccles, M.P. (2014). Theory-based predictors of multiple clinician behaviors in the management of diabetes. *Journal of Behavioral Medicine, 37*(4), 607-20.

Rantz, M.J., Popejoy, L., Petroski, G.F., Madsen, R.W., Mehr, D.R., … Maas, M. (2001). Randomized Clinical Trial of a Quality Improvement Intervention in Nursing Homes. *The Gerontologist, 41*(4), 525-538.

Ray, W.A., Federspiel, C.F., Schaffner, W. (1980). A study of antipsychotic drug use in nursing homes: epidemiologic evidence suggesting misuse. *American Journal of Public Health, 70*(5), 485-491.

Reed, J.E., McNicholas, C., Woodcock, T., Issen, L., Bell, D. (2014). Designing quality improvement initiatives: the action effect method, a structured approach to identifying and articulating programme theory. *BMJ quality & safety, 23*(12):1040-1048.

Rochon, P.A., Stukel, T.A., Bronskill, S.E., Gomes, T., Sykora, K. (2007). Variation in Nursing Home Antipsychotic Prescribing Rats. *Archives of Internal Medicine, 167,* 676-683.

Smeets, C.H.W., Smalbrugge, M., Zuidema, S.U., Derksen, E., de Vries, E., … Gerristen, D.L. (2014). Factors Related to Pyschotropic Drug Prescription for Neuropsychiatric Symptoms in Nursing Home Residents with Dementia. *JAMDA, 15,* 835-840.

Taylor J, Parmalee P, Brown H, Ouslander J. Falls response in: The falls management program: a quality improvement initiative for nursing facilities. Agency for Healthcare Research and Quality: Rickville, MD; 2005 [accessed May 27 2016]. *Available at: http://www.ahrq.gov/professionals/systems/long-term-care/resources/injuries/fallspx/index.html*

Tinetti M, Liu W, Ginter S. Mechanical restraint use and fall-related injuries among residents of skilled nursing facilities. Ann Intern Med 1992; 116(5):369–74.

Yin, R.K. (2009). *Case study research: Design and methods.* 4th ed. Los Angeles, CA: Sage publications.
