# Supplementary material for: Effect of Academic Detailing on Promoting Appropriate Prescribing of Antipsychotic Medication in Nursing Homes: A Cluster Randomized Clinical Trial
Source: JAMA Netw Open. 2020 May 26;3(5):e205724. doi: 10.1001/jamanetworkopen.2020.5724 (PMC7251442; doi:10.1001/jamanetworkopen.2020.5724)
Supplement: Supplement 2. — eTable 1. Description of Measured Variables and Data Source eTable 2. Description of Interactions eTable 3. Other Prescription Drugs Dispensed in Past 14 Days eTable 4. Discontinuation of Antipsychotic Among Residents Living in Nursing Homes at Baseline eTable 5. Summary of Intervention using TiDieR Suggested Categories [file jamanetwopen-3-e205724-s002.pdf]

Supplementary Online Content

Tadrous M, Fung K, Desveaux L, et al. Effect of academic detailing on promoting appropriate prescribing of antipsychotic medication in nursing homes: a cluster-randomized trial. *JAMA Netw Open.* 2020;3(5):e205724. doi:10.1001/jamanetworkopen.2020.5724

eTable 1. Description of Measured Variables and Data Source

eTable 2. Description of Interactions

eTable 3. Other Prescription Drugs Dispensed in Past 14 Days

eTable 4. Discontinuation of Antipsychotic Among Residents Living in Nursing Homes at Baseline

eTable 5. Summary of Intervention using TiDieR Suggested Categories

This supplementary material has been provided by the authors to give readers additional information about their work.

© 2020 Tadrous M et al. *JAMA Network Open.*

eTable 1. Description of Measured Variables and Data Source

Data Source	Variables
**Ontario Drug Benefits (ODB)**	• Continious Antipsychotic dispensing<br>• Daily Antipsychotic Dose<br>• Other medication use (ex. Benzodiazepine, antidepressant, acetomenophen)<br>• Number of concurrent drugs<br>•
**Canadian Institute for Health Information (CIHI) (NACRS and DAD)***	• Any hospitilization or emergency department visits<br>▪ Non-elective hospitilizations<br>▪ Unplanned ED visits<br>• Charlson Comorbidity score
**Ontario Health Insurance Plan (OHIP)**	• Dementia Diagnosis<br>• Psychiatrist visits<br>• Geriatrian visits<br>• Neugologist visits
**Continuing Care Reporting System (CCRS)**	• Dementia Diagnosis<br>• Psychosis<br>• Daily antipsychotic use<br>• Falls Assessments<br>• Clinical Scores<br>▪ Activities of Daily Living (ADl)<br>▪ Aggressive Behaviour Scale (ABS)<br>▪ Pain assessment<br>▪ Depression Rating Scle (DRS)
**Registered Person Database (RPDB)**	• Age<br>• Sex

© 2020 Tadrous M et al. *JAMA Network Open.*

eTable 2. Description of Interactions

Measure	Total
Total Number of providers engaged (includes duplicate visits)	**336**
Average number of visits per home (range)	**8.2 (0-36)**
Total Number of presentations and meetings	**112**
Average number of presentations or meetings per home (Range)	**6.2 (1-14)**

© 2020 Tadrous M et al. *JAMA Network Open.*

eTable 3. Other Prescription Drugs Dispensed in Past 14 Days

	Educational Outreach (EO)					Usual Care (UC)				
Time	Baseline	3	6	9	12	Baseline	3	6	9	12
**Number of Resident (n)**	**2,303**	**2,342**	**2,308**	**2,331**	**2,321**	**3,060**	**3,064**	**3,022**	**3,089**	**3,091**
**Any antidepressants (n,%)**	1,241 (53.9%)	1,246 (53.2%)	1,256 (54.4%)	1,242 (53.3%)	1,258 (54.2%)	1,635 (53.4%)	1,652 (53.9%)	1,635 (54.1%)	1,674 (54.2%)	1,666 (53.9%)
**Trazadone**	392 (17.0%)	371 (15.8%)	390 (16.9%)	362 (15.5%)	381 (16.4%)	551 (18.0%)	566 (18.5%)	548 (18.1%)	549 (17.8%)	553 (17.9%)
**TCA**	53 (2.3%)	51 (2.2%)	44 (1.9%)	46 (2.0%)	41 (1.8%)	77 (2.5%)	85 (2.8%)	73 (2.4%)	71 (2.3%)	67 (2.2%)
**Other**	1,060 (46.0%)	1,062 (45.3%)	1,078 (46.7%)	1,076 (46.2%)	1,079 (46.5%)	1,370 (44.8%)	1,379 (45.0%)	1,388 (45.9%)	1,435 (46.5%)	1,418 (45.9%)
**Benzodiazepine**	211 (9.2%)	225 (9.6%)	225 (9.7%)	220 (9.4%)	213 (9.2%)	347 (11.3%)	371 (12.1%)	310 (10.3%)	340 (11.0%)	326 (10.5%)
**Mood stabilizer**	261 (11.3%)	273 (11.7%)	273 (11.8%)	272 (11.7%)	279 (12.0%)	400 (13.1%)	403 (13.2%)	395 (13.1%)	411 (13.3%)	431 (13.9%)
**Acetaminophen**	1,040 (45.2%)	1,077 (46.0%)	1,039 (45.0%)	1,083 (46.5%)	1,102 (47.5%)	1,366 (44.6%)	1,385 (45.2%)	1,376 (45.5%)	1,368 (44.3%)	1,416 (45.8%)

© 2020 Tadrous M et al. *JAMA Network Open.*

eTable 4. Discontinuation of Antipsychotic Among Residents Living in Nursing Homes at Baseline

	Educational Outreach (EO)				Usual Care (UC)			
**Times**	**Baseline**	**3**	**6**	**9**	**Baseline**	**3**	**6**	**9**
**Residents on antipsychotics (N)**	**310**	**236**	**291**	**295**	**447**	**376**	**433**	**420**
3-month Follow-up	79 (25.5%)				112 (25.1%)			
6-month Follow-up	17 (5.5%)	20 (8.5%)			34 (7.6%)	35 (9.3%)		
9-month Follow-up	41 (13.2%)	42 (17.8%)	42 (14.4%)		30 (6.7%)	38 (10.1%)	36 (8.3%)	
12-month Follow-up	21 (6.8%)	25 (10.6%)	24 (8.2%)	23 (7.8%)	25 (5.6%)	31 (8.2%)	29 (6.7%)	28 (6.7%)
No Discontinuation	152 (49.0%)	149 (63.1%)	147 (50.5%)	147 (49.8%)	246 (55.0%)	272 (72.3%)	266 (61.4%)	265 (63.1%)

© 2020 Tadrous M et al. *JAMA Network Open.*

eTable 5. Summary of Intervention using TiDieR Suggested Categories

**Item**
**BRIEF NAME**
Academic detailing to promote appropriate prescribing of antipsychotics in nursing homes
**WHY**
To identify the real-world effectiveness of an academic detailing intervention in nursing homes across Ontario targeting appropriate prescribing of antipsychotics and the management of Behavioural and Psychological Symptoms of Dementia.
**WHAT**
Intervention:<br>• Academic detailing is a method of educational outreach which leverages one-on-one interactions to communicate evidence-based information to clinicians.<br>• Academic Detailers had direct and ongoing contact with the nursing homes including administrators, providers, and staff from the time of launch through to program completion.
**WHO PROVIDED**
• The intervention was delivered by health professionals (e.g., nurses or pharmacists) who arranged meetings (with administrators, physicians, pharmacists, nurses, and support workers), presentations, group visits (2–6 providers), and one-on-one visits (traditional academic detailing visits).
**HOW and WHERE**
• Detailers provided tailored information to help support nursing home clinical staff to address challenges and opportunities for improving prescribing of antipsychotics, providing additional materials and resources as needed.<br>• They also responded to questions via email or phone.<br>• Detailers provided tailored information to help support nursing home clinical staff to address challenges and opportunities for improving prescribing of antipsychotics, providing additional materials and resources as needed.

© 2020 Tadrous M et al. *JAMA Network Open.*

• Materials can be viewed here: https://cep.health/clinical-products/antipsychotics-and-dementia-primary-care-edition/
**WHEN and How Much**
When: Academic Detailing was launched in October 2015<br><br>How much: See Supplemental Appendix 2.
**TAILORING**
• Given the diverse target audiences that will likely be engaged within the homes, the Academic Detailer worked to understand the context, barriers, capacity, and needs of each home to ensure the service being provided by the detailer was relevant.

© 2020 Tadrous M et al. *JAMA Network Open.*
