# Supplementary material for: Effect of Academic Detailing on Promoting Appropriate Prescribing of Antipsychotic Medication in Nursing Homes: A Cluster Randomized Clinical Trial
Source: JAMA Netw Open. 2020 May 26;3(5):e205724. doi: 10.1001/jamanetworkopen.2020.5724 (PMC7251442; doi:10.1001/jamanetworkopen.2020.5724)
Supplement: Supplement 3. — Data Sharing Statement [file jamanetwopen-3-e205724-s003.pdf]

Data Sharing Statement

Tadrous. Effect of Academic Detailing on Promoting Appropriate Prescribing of Antipsychotic Medication in Nursing Homes. *JAMA Netw Open*. Published May 26, 2020. 10.1001/jamanetworkopen.2020.5724

Data
Data available: No

Additional Information
Explanation for why data not available: We used linked healthcare administrative data. Current regulations in the province of Ontario do not allow the public sharing of this data.
